# Informal Child Care and Adolescent Psychological Well-Being: Hong Kong’s “Children of 1997” Birth Cohort

**DOI:** 10.1371/journal.pone.0120116

**Published:** 2015-03-17

**Authors:** Cherry Y. Leung, Gabriel M. Leung, C. Mary Schooling

**Affiliations:** 1 School of Public Health, Li Ka Shing Faculty of Medicine, The University of Hong Kong, Hong Kong SAR, People’s Republic of China; 2 CUNY School of Public Health and Hunter College, New York, United States of America; Merced, UNITED STATES

## Abstract

**Background:**

Informal child care (child care by untrained family members, relatives or employees in the home) in Western populations is often associated with poorer psychological well-being, which may be confounded by socioeconomic position. We examined the association of informal child care, common in non-Western settings, with adolescent psychological well-being, using Hong Kong’s Chinese “Children of 1997” birth cohort.

**Methods:**

Multivariable linear regression was used to examine the adjusted associations of informal child care (at 0.5, 3, 5 and 11 years) with parent-reported Rutter score for child behavior at 11 years, self-reported Culture-Free Self-Esteem Inventories score at 11 years and self-reported Patient Health Questionnaire-9 depressive symptom score at 13 years. Model comparisons were used to identify the best representation of child care, in terms of a critical period of exposure to informal child care (independent variable) at a specific age, combination of exposures to informal child care at several ages or an accumulation of exposures to informal child care.

**Results:**

Child care was not associated with behavioral problems. A model considering child care at 3 years best represented the association of child care with self-esteem while a model considering child care at 5 years best represented the association of child care with depressive symptoms. Informal child care at 3 years was associated with lower self-esteem (-0.70, 95% confidence interval (CI) -1.26 to -0.14). Informal child care at 5 years was associated with more depressive symptoms (0.45, 95% CI 0.17 to 0.73).

**Conclusion:**

In a developed non-Western setting, informal child care was associated with lower self-esteem and more depressive symptoms.

## Introduction

The use of child care is increasing, especially with the surge of employment among women since the 1970s. In Western countries, such as the United States (U.S.), the likelihood of women working during their child’s infancy is the same as when they have an older preschooler [1]. Infancy and early childhood are a crucial age for neurological development. For example, the frontal cortex, where attention, behavior and judgment are controlled, matures most around 2 to 3.5 years [2,3]. Experiences in infancy and early childhood may have long-term impacts on cognition and psychological well-being [4,5]. Psychological well-being is integral to overall health and well-being and should be treated with the same priority as physical health [6,7]; 9.8% of adults are projected to be diagnosed with depression in high income countries by 2030 according to the World Health Organization (WHO) [8]. Poor adolescent psychological well-being, including low self-esteem and depressive symptoms, may presage depression in adulthood [9,10]. Globally, 20% of adolescents are diagnosed with mental illnesses [11], 10–15% with behavioral problems [12], 15–25% with low self-esteem [13,14] and 3–5% with depression [15].

Research into the association of child care with adolescent psychological well-being has been conducted from a number of perspectives mainly in Western settings. Most research has focused on investigating the role of maternal employment in behavior and mental health [16–18]. Some research has examined the role of formal child care (child care by trained and untrained caregivers in school or a child care center) [17,19,20]. Other research has examined the role of any type of child care without distinguishing between formal and informal child care (child care by untrained family members, relatives or employees in the home) [21–23] in Western settings. Only a few have examined informal care [24,25] as the sole exposure (or independent variable). Non-relative care (non-maternal care excluding grandparents and fathers) was associated with greater risk taking and impulsivity at age 15 [23] whereas maternal employment was found to be the main factor associated with poor cognitive and behavioral outcomes in early childhood [17]. One study concerning grandparent care found an association with greater hyperactivity and peer difficulties [25]. In the West, both informal child care and adolescent psychological well-being are socially patterned, such that low socioeconomic position (SEP) is associated with informal care [26–28] and poorer psychological well-being [28,29]. Thus, it is unclear whether observed associations are the result of child care or due to residual confounding arising from an association of low SEP with both informal child care and poor psychological well-being which has not been completely accounted for in the analysis. Informal care is common for children under age 5, estimated at 33 to 53% in the U.S. [27] and 45 to 50% in Britain [30], yet there is limited research focusing on how informal care is related to adolescent psychological well-being.

While there is growing body of evidence about formal child care in Western populations, less consideration has been given to the informal child care common in the rest of the global population, such as Asia, where care is often sought from informal caregivers such as grandparents, uncles, aunts or in-home employed help (including caregivers living with the family) [31,32]. Hong Kong is an economically developed non-Western setting, with a similar social infrastructure and rates of mental illness (16.4% of Hong Kong adolescents are diagnosed with mental illness) [33] to Western Europe and North America but similar to the rest of the global population, especially that of Asia [34], in seeking informal child care. In addition, informal child care is not associated with low SEP in Hong Kong. As such, Hong Kong is an ideal setting to study informal child care because informal child care is common in Hong Kong. The unique attributes of our setting, Hong Kong, means that this study, taking advantage of a large population representative Hong Kong Chinese birth cohort “Children of 1997,” will clarify the role of informal child care at 6 months and 3, 5 and 11 years in psychological well-being in early adolescence (11 to 13 years).

## Methods

### Ethics statement

Since our participants are children, informed consent was obtained from the parents, next of kin, caretakers or guardians (informants) on behalf of the participants by completing the questionnaire at enrollment as approved by The University of Hong Kong Medical Faculty Ethics Committee. Ethical approval for further studies was obtained from the University of Hong Kong-Hospital Authority Hong Kong West Cluster, Joint Institutional Review Board and/or the Ethics Committee of the Department of Health, Government of the Hong Kong SAR as appropriate.

### Participants

Hong Kong’s “Children of 1997” birth cohort is a population representative Chinese birth cohort (n = 8,327) that covered 88.0% of all births in Hong Kong from April 1, 1997 to May 31, 1997, and has been described in detail elsewhere [35]. The study was initially established to investigate the effects of secondhand smoke exposure on infant health. Families were recruited at the first postnatal visit to one of the 49 Maternal and Child Health Centres in Hong Kong, which parents of all newborns were encouraged to attend. Characteristics obtained using a self-administered questionnaire in Chinese at recruitment and subsequent routine visits included maternal and birth characteristics, parental education and early life exposures, such as breastfeeding. Passive follow-up via record linkage was instituted in 2005 to obtain routinely collected information including bi-annual assessments of emotional and behavioral problems using the Revised Parent’s Rutter Scales in Chinese [grade 2, 4 and 6 (i.e., ages 7–8, 9–10 and 11–12 years)] and self-esteem using Form A of the Culture-Free Self-Esteem Inventories for Children by Battle in Chinese [grade 4 (age 9–10 years) onwards] from the Student Health Service (SHS), Department of Health, which provides free annual check-ups for all school students. Active follow-up via direct contact was instituted in 2007, including postal surveys. Survey I, sent in July 2008, then re-sent a second and third time as necessary to non-respondents over the following 9 months, included questions on child care. Survey II, including the Patient Health Questionnaire-9 (PHQ-9), was first sent in February 2010 then re-sent a second and third time as necessary over the following 5 months. Non-responses were followed-up via two waves of telephone interviews from November 2010 to April 2011 and from July 2011 to June 2012, and during pilot studies for in-person follow-up (June to August 2011 and June to August 2012). A summary of information collection regarding type of child care and measures of psychological well-being is shown in Table 1.

**Table 1:** Collection of Information Regarding Child Care and Psychological Well-Being in Hong Kong’s “Children of 1997 Birth Cohort”.

Information	Source	Instrument	Provided by	Child’s age^a^
Type of child care at 0.5, 3, 5 and 11 years	Survey I	Survey question	Parents	11 years
Emotional and behavioral problems	SHS	Revised Parent’s Rutter Scales	Parents	9-<13 years
Self-esteem	SHS	Form A of the Culture-Free Self Esteem Inventories	Child	9-<13 years
Depressive symptoms	Survey II	Patient health questionnaire-9	Child	12–15 years

Abbreviation: SHS, Student Health Service.

^a^The child’s age when the information was collected.

### Informal child care exposure (independent variables)

Our primary exposures (independent variables) were exposure to informal child care at 6 months, 3 years, 5 years and 11 years, considered as separate exposures (independent variables) and as an accumulation of exposures (independent variables). Type of child care was obtained from this question in Survey I: ‘Who was your child’s main caregiver at the following different ages: 6 months, 3 years, 5 years, and now (11 years)?’ with responses specified as ‘parent’, ‘grandparent’, ‘domestic helper’ (in-home employed help) and ‘other.’ ‘Other’ included a fill-in-the-blank portion. Most ‘other’ responses were care by other relatives with very little formal care (daycare, nursery and crèche, n = 10 at 6 months and n = 12 at 3 months). Child care in our study was categorized in two ways. First, at each of 6 months and 3, 5 and 11 years, as *parental* if the child was cared for by either parent or *informal* if provided by grandparents, in-home employed help, or other relatives in the child’s own home. Those reported to have more than one main caregiver were categorized as *informal* because parental and informal care were mutually exclusive in this study. Second, *informal* was further categorized as grandparent, in-home employed help or other.

## Outcomes

### Emotional and behavioral problems

Emotional and behavioral problems from 9 to <13 years (mean age 11 years) were assessed from the Revised Parent’s Rutter Scales in Chinese for parents [36,37]. Cohen’s kappa to test for item agreement between English and Chinese was excellent (0.81) while the correlation for the scales were high (0.94) [37]; furthermore, the discriminative power for behavioral problems was good [36]. The scales consist of a set of 31 items describing emotional and behavioral difficulties, with each item scored 0 for *does not apply*, 1 for *applies somewhat* or 2 for *certainly applies*. A total score was calculated, where a higher score indicated more emotional and behavioral problems.

### Self-esteem

Self-esteem at from 9 to <13 years (mean age 11 years) was assessed from the Form A of the Culture-Free Self Esteem Inventories (SEI) [38] in Chinese for children. The SEI correlates well with other widely used instruments, including Stanley Coopersmith’s Self Esteem Inventory (1967); correlations ranged from 0.72 to 0.93 among primary school students [39], as was found with a study on Chinese children in Hong Kong [40]. A total score (50 items), was calculated, where a lower score indicated lower self-esteem. We excluded unreliable scores with a lie subscale (of the SEI) of 2 or less (n = 158), although a lie subscale of 5 or more has been used to indicate a lack of defensiveness and a reliable self-esteem inventories [38].

### Depressive symptoms

Depressive symptoms from 12 to ≤15 years (mean age 13 years) were assessed from the PHQ-9 [41] in Chinese as part of Survey II. The PHQ-9 scale has good sensitivity and specificity in detecting depression in youth [42], is an effective screening tool for the risk of depression among adolescents [43] and has been validated in Chinese adolescents [44]. The scale consists of a set of 9 items describing symptoms and functional impairment, with each item scored 0 for *not at all*, 1 for *several days*, 2 for *more than half the days* or 3 for *nearly everyday*. A total PHQ-9 score was calculated, where a higher score indicated more depressive symptoms.

### Covariates

Potential confounders considered, as classified in Table 2, were sex, mother’s birthplace, highest parental education, highest parental occupation at birth, monthly household income per head at birth, maternal age at birth and parity. These were all self-reported at recruitment, with contradictory, missing or incomplete information clarified in subsequent data collection exercises. Specifically, information from subsequently collected information has been used to correct: 1) sex in a very few cases, where it appeared to be discrepant, 2) to obtain education in a few cases where it was missing from the baseline survey and 3) to improve the classification of mother’s birthplace because the question on the baseline survey was not entirely unambiguous.

**Table 2:** Characteristics of Respondents (n = 3,679) and Non-Respondents (n = 4,257) to Survey I in 2008–2009 of Hong Kong’s “Children of 1997 Birth Cohort” (Available Case Analysis).

	Respondents	Non-Respondents	
Characteristics	*n* (column %)	*n* (column %)	Cohen effect size^a^
**Sex**			
Girl	1,888 (51.3)	1,862 (43.8)	0.15
Boy	1,791 (48.7)	2,394 (56.3)	
**Mother's birthplace**			
Hong Kong	2,217 (60.5)	2,180 (62.0)	0.03
Non-Hong Kong	1,440 (39.5)	1,413 (38.0)	
**Highest parental education**			
Grade 9 or below	1,054 (28.7)	1,339 (32.8)	0.11
Grade 10–11	1,574 (42.8)	1,755(43.0)	
Grade 12 or above	1,049 (28.5)	991(24.2)	
**Highest parental occupation at birth**			
I (professional)	847 (26.4)	807 (22.3)	0.09
II (managerial)	473 (14.7)	566 (15.6)	
IIINM (non-manual skilled)	935 (29.2)	1,057 (29.2)	
IIIM (manual skilled)	531 (16.6)	661 (18.3)	
IV(semi-skilled)	322 (10.1)	389(10.7)	
V (unskilled)	95 (3.0)	141 (3.9)	
**Monthly household income per head at birth in quintiles, dollars** ^b^ **[mean (SD)]**			
1^st^ quintile [1,801 (428)],	602 (18.5)	815 (21.9)	0.12
2^nd^ quintile [3,036 (428)]	636 (19.5)	800 (21.4)	
3^rd^ quintile [4,715 (516)],	642 (19.7)	746 (20.0)	
4^th^ quintile [7,180, (913)],	679 (20.8)	690 (18.5)	
5^th^ quintile [15,091 (11,390)]	700 (21.5)	680 (18.2)	
**Maternal age at birth**			
24 or below	348 (9.5)	611 (15.0)	0.16
25–29	1,137 (30.9)	1,276 (31.3)	
30–34	1,466 (39.9)	1,502 (36.9)	
35 or above	724 (19.7)	685 (16.8)	
**Parity**			
1	1,714 (48.0)	1,929 (47.2)	0.04
2	1,490 (41.8)	1,674 (41.0)	
3 or above	363 (10.2)	483 (11.8)	

Abbreviation: n, number; SD, standard deviation.

^a^Cohen effect sizes have three levels: 0.1 for small, 0.3 for medium and 0.5 for large.

^b^In Hong Kong dollars (HK $7.80 = US $1).

### Statistical analysis

We compared the characteristics of respondents and non-respondents to Survey I using Cohen effect sizes. Given exposures to child care are correlated and the role of child care at different ages is not well-defined, we used model comparisons, as in a previous study [45], to ascertain the best way of using the information about child care. We compared models representing child care in terms of: 1) a critical period (i.e., exposure at 6 months, 3 years, 5 years or 11 years), 2) independent exposures at each age considered together and 3) an accumulation of exposures (number of exposures to informal care treated as continuous). We used model fit, from the Akaike Information Criterion (AIC), to compare these different representations of child care [46] with behavioral problems, self-esteem and depressive symptoms. A lower AIC indicates a better fitting model. By determining the best fitting model through using the AIC, we can determine whether exposure to informal child care at a certain critical period or at a combination of periods best fits the data. We then used multivariable linear regression to estimate the adjusted association of our independent variable, informal child care exposure, with Rutter, SEI, and depressive symptom scores from which single estimated beta-coefficients with 95% confidence intervals (CIs) are presented. The potential confounders, age of assessment and survey mode for depression were included in the models. PHQ-9 was obtained by postal questionnaire, telephone interview and in-person interview, hence the inclusion of survey mode in the relevant models. As a sensitivity analysis, we also adjusted for maternal depression at the time the participant was 13 years old, which was obtained by maternal self-report from Survey II.

Given that we have a population representative birth cohort with comprehensive baseline data, we used a combination of inverse probability weighting and multiple imputation [47]. Inverse probability weights were estimated using logistic regression with variables that could significantly predict the pattern of missingness for Survey I, including highest parental education, monthly household income per head, highest parental occupation, housing type, maternal age, maternal place of birth, parity and child’s sex, to account for potential differences between those who responded to Survey I and those who did not. We then used multiple imputation to predict missing values of the independent variables and confounders incorporating data on the outcomes (three behavioral and psychological instruments), independent variables, confounders and other factors potentially associated with child care. We summarized the results from 10 imputed datasets into single estimated beta-coefficients with CIs and AIC values adjusted for missing data uncertainty using the ‘Hmisc’ package in R 3.0.1 (R Development Care Team, Vienna, Austria). We also performed a complete case analysis, which excludes individuals with missing data from the analysis, for comparison using Stata version 10 (Stata Corp, College station, Texas).

## Results

Of the original 8,327 cohort members, 26 (0.3%) had permanently withdrawn. Of the remaining 8,301, 7,933 were potentially contactable in 2010–2012. The follow-up rates for Rutter score for child behavior was 71% (n = 5,598) and Culture-Free Self-Esteem Inventory score was 87% (n = 6,937); the response rate for PHQ-9 depressive symptom score was 73% (n = 5,797).

Characteristics of respondents compared to non-respondents to Survey I are shown in Table 2. The respondents were slightly different from non-respondents, but the Cohen effect sizes were small (<0.2) [48]. Of the respondents, nearly 50% of participants had informal care at 6 months (49.7%) and 3 years (46.7%). Use of informal care was less common at older ages, 41.0% at 5 years and 23.9% at 11 years. Informal care at all ages was more common among children from families of higher SEP (assessed from parental education, household income per head or parental occupation), with fewer siblings and with Hong Kong born mothers (Table 3). Care by in-home employed help, rather than grandparents, was more common for higher SEP children. Care by in-home employed help, rather than grandparents, was more common at older ages, for children with fewer siblings and for children with Hong Kong born mothers.

**Table 3:** Baseline Characteristics by Child Care from Hong Kong’s “Children of 1997 Birth Cohort” (Available Case Analysis).

	6 Months			3 Years			5 Years			11 Years		
Characteristics	*n*	% of *n* in informal care	χ^2^ *P*-Value	*n*	% of *n* in informal care	χ^2^ *P*-Value	*n*	% of *n* in informal care	χ^2^ *P*-Value	*n*	% of *n* in informal care	χ^2^ *P*-Value
**Sex**												
Girl	1,852	48.1	0.061	1,852	44.6	0.013	1,852	39.2	0.034	1859	24.7	0.257
Boy	1,753	51.2		1,750	48.7		1,753	42.7		1759	23.1	
**Mother's birthplace**												
Hong Kong	2,184	61.7	<0.001	2,180	57,8	<0.001	2,183	51.0	<0.001	2191	29.4	<0.001
Non-Hong Kong	1,412	31.0		1,413	29.4		1,413	25.5		1418	15.5	
**Highest parental education**												
Grade 9 or below	1,019	22.8		1,016	20.1	<0.001	1,019	17.6	<0.001	1027	10.3	<0.001
Grade 10–11	1,548	54.4		1,548	50.7		1,548	44.4		1549	24.8	
Grade 12 or above	1,038	68.9		1,038	66.4		1,038	59.6		1042	36.0	
**Highest parental occupation at birth**												
I (professional)	837	73.0	<0.001	836	67.9	<0.001	837	61.8	<0.001	840	35.6	<0.001
II (managerial)	463	58.3		464	59.5		464	51.3		465	32.3	
IIINM (non-manual skilled)	917	60.9		917	55.8		915	48.1		916	27.0	
IIIM (manual skilled)	516	22.5		515	16.5		515	15.7		518	8.7	
IV(semi-skilled)	317	20.5		315	20.6		318	18.6		319	12.5	
V (unskilled)	90	17.8		90	18.9		90	11.1		91	3.3	
**Monthly household income per head at birth in quintiles** ^a^, **dollars** ^b^ **[mean (SD)]**												
1^st^ quintile	587	24.4	<0.001	585	22.6	<0.001	588	18.4	<0.001	587	10.1	<0.001
2^nd^ quintile	615	27.3		614	25.2		614	23.5		620	13.2	
3^rd^ quintile	630	44.6		629	41.3		630	37.1		633	20.9	
4^th^ quintile	667	70.9		668	63.8		667	55.9		670	30.5	
5^th^ quintile	692	78.9		692	75.7		692	67.5		692	42.1	
**Maternal age at birth**												
24 or below	343	42.6	<0.001	345	42.9	<0.001	344	38.1	<0.001	345	27.3	<0.001
25–29	1,118	48.4		1,118	45.6		1,120	40.2		1123	24.9	
30–34	1,440	54.1		1,436	50.7		1,437	45.0		1441	25.3	
35 or above	702	46.0		701	41.5		702	35.0		707	17.8	
**Parity**												
1	1,687	59.8	<0.001	1,689	56.9	<0.001	1,687	50.7	<0.001	1695	32.2	<0.001
2	1,461	45.6		1,455	42.0		1,460	36.4		1464	19.3	
3 or above	349	25.2		350	47.6		350	21.1		351	8.6	

Abbreviation: n, number; SD, standard deviation.

^a^6 months: 1^st^ quintile [1,801 (425)], 2^nd^ quintile [3,037 (429)], 3^rd^ quintile [4,714 (517)], 4^th^ quintile [7,182 (912)], 5^th^ quintile [15,087 (11,382)]; 3 years: 1^st^ quintile [1,798 (428)], 2^nd^ quintile [3,037 (429)], 3^rd^ quintile [4,736 (569)], 4^th^ quintile [7,208 (899)], 5^th^ quintile [15,087 (11,382)]; 5 years: 1^st^ quintile [1,799 (427)], 2^nd^ quintile [3,038 (428)], 3^rd^ quintile [4,715 (517)], 4^th^ quintile [7,181 (913)], 5^th^ quintile [15,087 (11,382)]; 11 years: 1^st^ quintile [1,801 (428)], 2^nd^ quintile [3,036 (428)], 3^rd^ quintile [4,715 (515)], 4^th^ quintile [7,180 (913)], 5^th^ quintile [15,091 (11,391)].

^b^In Hong Kong dollars (HK $7.80 = US $1).

Table 4 shows the best fitting model for exposures to informal child care for each measure of psychological well-being, adjusted for sex, mother’s birthplace, highest parental education, highest parental occupation, monthly household income per head, maternal age at birth, parity, age of assessment and survey mode (PHQ-9 scores). Informal care at 11 years was the best fitting model for the Rutter score, informal care at 3 years was the best fitting model for the self-esteem score and informal care at 5 years was the best fitting model for the PHQ-9 score.

**Table 4:** AIC Values from Adjusted^a^ Regression Models for Measures of Psychological Well-Being Using Different Representations of Child Care (After Inverse Probability Weighting with Multiple Imputation).

	Measure of Psychological Well-Being		
Child Care at:	Rutter score	Self-esteem score	PHQ-9 score
6 months	17370.8	22382.8	17029.01
3 years	17370.9	**22380.7** ^b^	17027.81
5 years	17370.7	22384.4	**17022.7** ^b^
11 years	**17368.1** ^b^	22386.1	17031.93
6 months and 3 years	17372.8	22382.2	17029.02
6 months and 5 years	17372.6	22384.2	17024.21
6 months and 11 years	17370.1	22384.5	17030.25
3 and 5 years	17372.5	22382.5	17024.65
3 and 11 years	17369.8	22382.7	17029.58
5 and 11 years	17369.9	22386.4	17024.48
6 months and 3, 5 years	17374.4	22384.0	17025.94
6 months and 3, 11 years	17371.7	22384.2	17030.8
6 months and 5, 11 years	17371.8	22386.2	17025.99
3, 5, and 11 years	17371.8	22384.4	17026.42
6 months and 3, 5, 11 years	17373.6	22385.9	17027.71
Exposure to care (continuous)	17370.3	22381.8	17024.97

Abbreviation: AIC, Akaike Information Criterion.

^a^Adjusted for sex, mother’s birthplace, highest parental education, highest parental occupation at birth, household income per head at birth, maternal age at birth, parity, age of assessment and survey mode (PHQ-9 scores).

^b^Lowest AIC represents the best fitting model.

Based on the best fitting models that give the age at which child care had the most impact on each measure of psychological well-being, we further examined these specific associations of informal care with psychological well-being. Associations, adjusted for sex, mother’s birthplace, highest parental education, highest parental occupation, monthly household income per head, maternal age at birth, parity, age of assessment and survey mode (PHQ-9 scores), using the best fitting models are shown in Table 5. Informal care was not associated with the Rutter score. However, informal care at 3 years, compared to parental care, was associated with a lower self-esteem score. Informal care at 5 years was associated with a higher PHQ-9 score; this association remained after further adjustment for maternal depression when the child was about 13 years (data not shown).

**Table 5:** Adjusted^a^ Association of Informal Care Compared to Parental Care with Psychological Well-Being (After Inverse Probability Weighting with Multiple Imputation).

Measure of Psychological Well-Being (Dependent Variable or Outcome)	Child Care Exposure (Independent Variable) Considered		
		β	95% CI
Rutter score	Parental care only	Ref	
	Informal care at 11 years	-0.51	(-1.05 to 0.04)
Self-esteem score	Parental care only	Ref	
	Informal care at 3 years	**-0.70**	**(-1.26 to -0.14)**
PHQ-9 score	Parental care only	Ref	
	Informal care at 5 years	**0.45**	**(0.17 to 0.73)**

Abbreviation: CI, confidence interval, Ref, reference.

^a^Adjusted for sex, mother’s birthplace, highest parental education, highest parental occupation at birth, household income per head at birth, maternal age at birth, parity, age of assessment and survey mode (PHQ-9 scores).

To elucidate the role of the provider of informal care, we examined the association of different types of informal care with the Rutter score, self-esteem score and PHQ-9 score (S1 Table). Exclusive grandparent care, compared to parental care, at 11 years was associated with a lower Rutter score. A lower self-esteem score was more marked for care by in-home employed help and others at 3 years. Similarly, the association of informal care at 5 years with a higher PHQ-9 score was more marked for care by in-home employed help and others.

As a sensitivity analysis, we also presented results from a complete case analysis adjusted for the same set of confounders (S2 and S3 Tables). The findings were similar to the results from inverse probability weighting with multiple imputation.

## Discussion

Using a large population representative birth cohort from the economically developed non-Western setting of Hong Kong where informal child care is common and associated with higher SEP, this study provides new evidence that informal child care, compared to parental care, during childhood is associated with some aspects of poorer adolescent psychological well-being. Informal child care, as compared to parental care, was not clearly associated with behavioral problems, although care by grandparents at 11 years was perhaps associated with better behavior. However, informal child care at 3 years was associated with lower self-esteem, mainly driven by care by in-home employed help, while informal care at 5 years was associated with more depressive symptoms, again mainly driven by exposure to in-home employed help.

Despite our population representative birth cohort, the study has limitations. First, the proportion of respondents for Survey I regarding child care was not high. While we performed a complete case analysis with similar results, our primary analysis employed a combination of inverse probability weighting with multiple imputation since complete case analysis requires more assumptions to be valid [47]. However, there are disadvantages with this method. Since inverse probability weighting only models the probability that an individual has complete data, multiple imputation is used to supplement this by modelling the joint distribution of missing data given the observed data [47]. When the percentage of missing data is high, multiple imputation of independent variables could bias towards the null while multiple imputation of confounders might generate residual confounding. Nonetheless, studies have shown that complete case analysis has more biased results [49]. Second, quality of child care, which may also be associated with child health outcomes [21], was not measured in our study nor was quantity of time spent with each particular caregiver. In Hong Kong, parents or grandparents can be present and overseeing care provided by in-home employed help. Third, maternal depression in infancy and toddlerhood are associated with child behavioral and adjustment problems, whether the depression is due to genetic transmission, observational learning or impaired parenting [50]. Maternal depression during infancy and toddlerhood was not measured. However, adjustment for maternal depression when the participant was 13 years made little difference (data not shown). Fourth, recall bias is a limitation. On the other hand, it is most likely non-differential, impairing precision rather than creating bias. Fifth, our study assessed emotional and behavioral problems, self-esteem and depressive symptoms using screening tools, which increases the chances of reporting bias especially when stereotypes and stigmas exist about mental health, instead of by clinical diagnosis. These questionnaires have been validated in Chinese populations as described in our methods [33,36,51]. Sixth, we used the AIC to compare models, which provides us with the best fitting model inferred from our data and is ideally used for large sample sizes as ours, but ultimately does not provide us with any information if a better model with care at other age groups or combination of age groups exists since it is not specified in our data [52]. Finally, given that formal child care is rarely used in Hong Kong, we could not assess the role of formal as compared to informal child care. Formal child care provided by professionals may have very different effects from informal child care.

There are several possible explanations for our observations. Informal caregivers may have less knowledge about promoting child development and psychological well-being because parents are the typical attendees at well-child primary care visits. Moreover, literacy may be an issue as employed helpers are usually from Southeast Asia. Informal caregivers may have less time to devote to the child as in-home employed caregivers are also usually responsible for running the household. Alternatively, the child may find it difficult to negotiate a number of different caregivers at an early age, given it may not be the same in-home employed help provider throughout childhood, with corresponding effects on self-esteem and vulnerability to depression. Bowlby’s ‘maternal deprivation’ hypothesis, that a child should only be attached to his/her mother during early life, has been disproved as this relationship can be compensated by an attachment to another primary figure [53]. However, while moderate shared caring of children is beneficial, negative immediate and long-term effects are seen when many adults are involved [53]. Better outcomes of psychological well-being as associated with consistent exposure to a particular caregiver, specifically parents and grandparents in our study, support this notion.

From a public health perspective, these results suggest that specific types of informal child care could have negative consequences on adolescent psychological well-being. Prevention could perhaps be achieved by interventions aimed at informal caregivers’ skills or alternatively at ensuring that informal caregivers have sufficient time and energy to devote to the child. Given the importance of psychological well-being and changing attitudes over time with the widespread use of informal care in non-Western settings, further investigation and experimentation could be worthwhile.

## Conclusion

In a developed non-Western setting where informal child care is common and associated with higher SEP, informal child care, perhaps particularly by in-home employed help, is prospectively associated with lower self-esteem and higher depressive symptom scores in adolescence. As such, our study suggests early life exposures, particularly the provision of informal child care, may be associated with subsequent psychological well-being.

## Supporting Information

S1 Table Adjusted^a^ Association of Informal Care by Provider Compared to Parental Care with Psychological Well-Being (After Inverse Probability Weighting with Multiple Imputation). (DOCX)

S2 Table Adjusted^a^ Association of Informal Care Compared to Parental Care with Psychological Well-Being (Complete Case Analysis). (DOCX)

S3 Table Adjusted^a^ Association of Informal Care with Psychological Well-Being by Provider Compared to Parental Care (Complete Case Analysis). (DOCX)
