# Supplementary material for: Informal Child Care and Adolescent Psychological Well-Being: Hong Kong’s “Children of 1997” Birth Cohort
Source: PLoS One. 2015 Mar 17;10(3):e0120116. doi: 10.1371/journal.pone.0120116 (PMC4363320; doi:10.1371/journal.pone.0120116)
Supplement: S1 Table — (DOCX) [file pone.0120116.s001.docx]

**Table S1. Adjusted^a^ Association of Informal Care by Provider Compared to Parental Care with Psychological Well-Being (After Inverse Probability Weighting with Multiple Imputation).**

| **Measure of Psychological Well-Being (Dependent Variable or Outcome)** | **Child Care Exposure (Independent Variable) Considered** | **Child Care by Provider** |  | |
| --- | --- | --- | --- | --- |
|  |  |  | β | 95% CI |
| Rutter score | Informal care at 11 years | Parental care only | Ref |  |
|  |  | All grandparent | **-1.26** | **(-2.09 to -0.43)** |
|  |  | All employed help | -0.03 | (-0.71 to 0.66) |
|  |  | All other | 0.04 | (-1.53 to 1.60) |
| Self-esteem score | Informal care at 3 years | Parental care only | Ref |  |
|  |  | All grandparent | -0.42 | (-1.14 to 0.30) |
|  |  | All employed help | **-0.86** | **(-1.58 to -0.15)** |
|  |  | All other | **-1.34** | **(-2.38 to -0.30)** |
|  |  |  |  |  |
| PHQ-9 score | Informal care at 5 years | Parental care only | Ref |  |
|  |  | All grandparent | 0.35 | (-0.01 to 0.73) |
|  |  | All employed help | **0.50** | **(0.15 to 0.84)** |
|  |  | All other | **0.73** | **(0.13 to 1.34)** |

Abbreviation: CI, confidence interval, Ref, reference.

^a^Adjusted for sex, mother’s birthplace, highest parental education, highest parental occupation at birth, household income per head at birth, maternal age at birth, parity, age of assessment and survey mode (PHQ-9 scores).
