# Supplementary material for: Informal Child Care and Adolescent Psychological Well-Being: Hong Kong’s “Children of 1997” Birth Cohort
Source: PLoS One. 2015 Mar 17;10(3):e0120116. doi: 10.1371/journal.pone.0120116 (PMC4363320; doi:10.1371/journal.pone.0120116)
Supplement: S2 Table — (DOCX) [file pone.0120116.s002.docx]

**Table S2. Adjusted^a^ Association of Informal Care Compared to Parental Care with Psychological Well-Being (Complete Case Analysis).**

| **Measure of Psychological Well-Being (Dependent Variable or Outcome)** | **Child Care Exposure (Independent Variable) Considered** |  | |
| --- | --- | --- | --- |
|  |  | β | 95% CI |
| Rutter score | Parental care only | Ref | |
|  | Informal care at 11 years | **-0.77** | **(-1.37 to -0.17)** |
| Self-esteem score | Parental care only | Ref |  |
|  | Informal care at 3 years | **-0.86** | **(-1.49 to -0.22)** |
| PHQ-9 score | Parental care only | Ref |  |
|  | Informal care at 5 years | **0.48** | **(0.17 to 0.78)** |

Abbreviation: CI, confidence interval, Ref, reference.

^a^Adjusted for sex, mother’s birthplace, highest parental education, highest parental occupation at birth, household income per head at birth, maternal age at birth, parity, age of assessment and survey mode (PHQ-9 scores).
